# Non-ulcerated Necrotizing Sialometaplasia: A case series

**DOI:** 10.4317/jced.63606

**Published:** 2026-02-26

**Authors:** Constanza Godoy-Latorre, Macarena Rodríguez-Luengo, Rolando Morales, Pablo Córdova, Maureen Marshall, René Martínez, Sven Eric Niklander

**Affiliations:** 1 Faculty of Dentistry, Universidad Andres Bello, Chile; 2 Department of Morphology, Faculty of Medicine, Universidad Andres Bello, Chile; 3 Unit of Oral Pathology and Oral Medicine, Faculty of Dentistry, Universidad Andres Bello, Chile

## Abstract

Necrotizing sialometaplasia is an uncommon, locally destructive inflammatory condition that most frequently affects the minor salivary glands of the palate, although salivary glands at other sites can also be affected. Its clinical presentation is generally characterized by the presence of a painful, ulcerated swelling, but in the early stages, it might not be ulcerated which makes its diagnosis very challenging. Although it is a benign, self-limited condition, care must be taken during diagnosis, as it can mimic malignant processes, both clinically and histologically. Here we report three uncommon cases of non-ulcerated necrotizing sialometaplasia with its clinical and histopathological characteristics.

## Introduction

Necrotizing sialometaplasia (NS) is an uncommon, non-neoplastic self-limiting lesion of the salivary glands, most frequently involving the minor salivary glands of the posterior hard palate (1). Its pathogenesis is attributed to ischemic infarction of salivary gland tissue caused by trauma or vascular compression of the surrounding area (2). The lesion typically progresses through five consecutive stages: infarction, sequestration, ulceration, repair, and healing (3). Reported predisposing factors include alcohol and tobacco use, upper respiratory infections, local anesthesia, arteriosclerosis, vasculitis and persistent vomiting (4 , 5). Although NS can occur at any age, it is most commonly observed in middle-aged males (2 , 4 , 5). Clinically, NS often manifests as a non-healing ulcer or ulcerated nodule, closely mimicking a benign or malignant neoplasm (6). Non-ulcerated presentations have also been documented, but these are uncommon and probably represent an early stage of the lesion. Histologically, NS is characterized by coagulative necrosis of glandular acini with preservation of lobular architecture and squamous metaplasia of the ductal epithelia. Pseudoepitheliomatous hyperplasia of the mucosal epithelium is also described during the reparative phase of the disease. This, in addition with the presence of squamous metaplasia of the ductal epithelium, may lead to the misdiagnosis of a malignant neoplasm, such as mucoepidermoid carcinoma or squamous cell carcinoma (3 , 4 , 7 , 8). Such diagnostic errors can result in unnecessary aggressive surgical interventions, which are not necessary for the treatment of NS given its self-healing nature (9). Reports of non-ulcerated NS are scarce in the literature, and distinguishing these cases from other conditions- both clinically and histopathologicallly- remains challenging (6). Here, we present three cases of non-ulcerated NS of the palate, detailing its clinical and histological characteristics.

## Case Report

**Figure 1:**
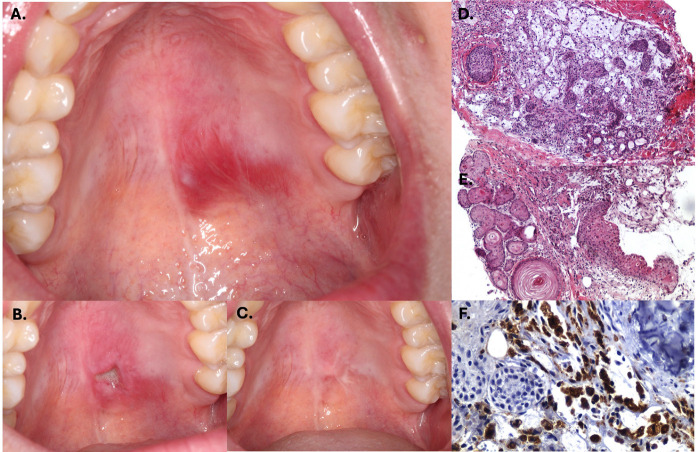
The lesion presented as a red swelling with superficial telangectasias (A.). Clinical appearance seven days post incisional biopsy (B.), and follow-up after 2 month (C.). Histopathological features showing well conserved necrotic acini (D.) with squamous metaplasia and keratin formation (E.). CD68 positive cells highlighting intense macrophagic infiltrate (F.).

Case 1: A 22-year-old female presented with an asymptomatic palatal swelling of three weeks' duration (Fig. 1), reportedly following food-related trauma. She had no relevant medical history.

1

**Figure 2:**
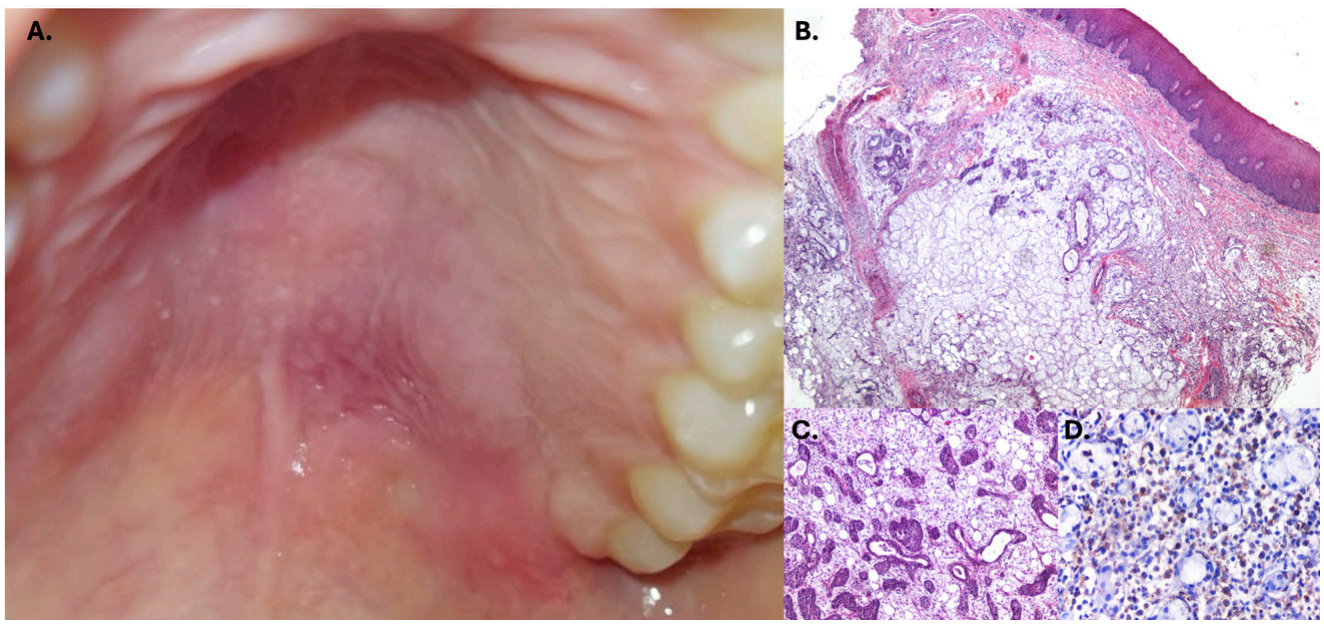
Initial presentation of the lesion as a mild erythematous swelling (A.). Fragment of palatal mucosa showing well-conserved necrotic acini (B.) with squamous metaplasia (C.), and an intense macrophagic infiltrate (CD68+ cells) among the necrotic acini (D.) and areas of mucin extravasation.

A malignant salivary gland tumor was initially suspected, and an incisional biopsy was performed. Histopathological examination revealed acinar necrosis, ductal squamous metaplasia, and foamy histiocytes (Fig. 1). A diagnosis of necrotizing sialometaplasia was established. The patient was treated with topical clobetasol, and the lesion resolved completely within two months (Fig. 1). Case 2: A 40-year-old female with no significant medical history presented with a painless palatal swelling of four weeks' duration. The patient did not associate the lesion to any factor (Fig. 2).

2

**Figure 3:**
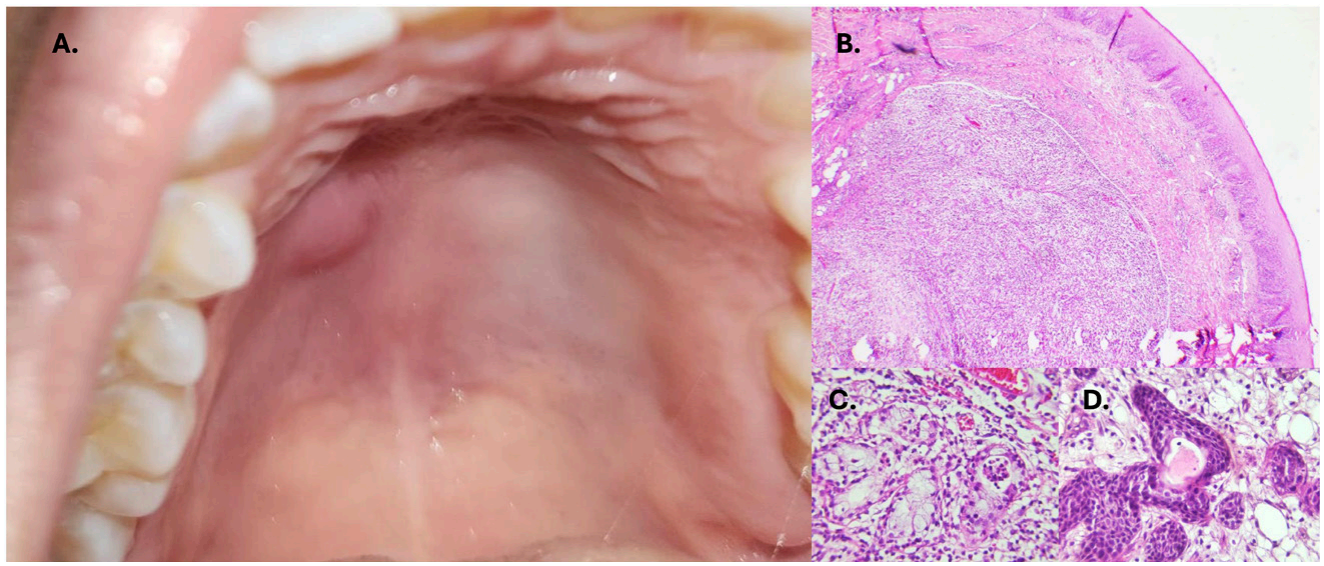
Unspecific swelling affecting the anterior part of the hard palate (A.). Well localized chronic inflammatory process (B.) with necrotic and remaining acini (C.) Areas of squamous metaplasia (D).

A malignant salivary gland neoplasm was suspected, prompting an incisional biopsy. Histopathological analysis revealed extensive acinar necrosis, ductal metaplasia, and numerous foamy histiocytes (Fig. 2). A diagnosis of necrotizing sialometaplasia was made. The patient was subsequently lost to follow-up. Case 3: A 36-year-old female presented with a painless palatal swelling of two weeks' duration. The patient did not report any attributed factor (Fig. 3).

3

She had no relevant medical history. On palpation, the lesion was soft and fluctuant. An incisional biopsy was performed to rule out a salivary gland neoplasm. Histopathological findings included localized inflammation surrounding necrotic acini and abundant histiocytes (Fig. 3). A diagnosis of necrotizing sialometaplasia was confirmed, and the lesion resolved spontaneously within six weeks.

## Discussion

NS is an uncommon disorder of the salivary glands, traditionally attributed to trauma, drugs that impair perfusion, or thrombotic events leading to infarction. This process ultimately results in ischemic necrosis of the salivary glands (2 , 10). Based on its pathophysiology, several potential risk factors have been proposed, including drug abuse, tobacco use, dental anesthesia on the affected area, and systemic conditions associated with increased thromboembolic risk. Additionally, multiple cases of NS have been reported in patients with bulimia, likely due to the irritating effects of gastric contents with low pH or microtrauma to the salivary gland stroma during repeated vomiting (2 , 11). Nevertheless, in most cases, the exact cause cannot be determined (12), as in two of the cases presented here. The diagnosis of NS can be challenging due to its overlapping clinical, anatomical, and histological features with other oral pathologies, particularly salivary gland neoplasms. Clinically, NS evolves over time: it often begins as a nodule or swelling covered by intact mucosa, which may appear purple, bluish, or erythematous (12). Approximately 81% of non-ulcerated lesions are associated with pain or tenderness (13). As the lesion progresses, a painful ulcer measuring several millimeters typically develops (6 , 12). In the three cases presented, the lesions appeared as asymptomatic swellings, with only one exhibiting erythema. They were located unilaterally on the hard palate-the most common site for NS (2 , 9). This initial presentation, followed by ulceration and pain, closely resembles the clinical features of malignant salivary gland tumors such as mucoepidermoid carcinoma (MEC), and adenoid cystic carcinoma. Squamous cell carcinoma should also be considered in the differential diagnosis (7). The histological diagnosis of NS can be challenging. Although the diagnostic criteria initially described by Abrams et al. remain unchanged; massive infarction, bland nuclear features, simultaneous metaplasia of the ducts and acini, prominence of inflammatory granulation tissue, and maintenance of the lobular structures (10), studies indicate that up to 21% of NS cases are misinterpreted as neoplastic lesions, often leading to unnecessary invasive procedures (8 , 9). Squamous ductal metaplasia, together with pseudoepitheliomatous hyperplasia of the surface epithelium are characteristic of the reparative phase of NS and may mimic features of salivary gland tumors such as mucoepidermoid carcinoma (MEC) (2 , 3 , 5 , 8). Chronic inflammatory cells are common to observe, and neutrophil infiltration is also frequent (3). Foamy macrophages can also be present especially in cases associated with mucin extravasation. One of our cases (case N°3) presented no metaplastic changes, probably due its short evolution (2 weeks). Key distinguishing features include the absence of cytologic atypia, pleomorphism, hyperchromatism (although mild atypia, pleomorphism and hyperchromatism might be observable in more inflamed lesions) and stromal invasion, combined with preservation of the normal lobular architecture of salivary glands (8). Recognizing these criteria is essential to avoid misdiagnosis and overtreatment. Cases of necrotizing sialometaplasia (NS) occurring in conjunction with salivary gland neoplasms have been documented. Zhurakivska et al. reported that 25% of NS cases in minor salivary glands were associated with an underlying salivary gland tumor, likely due to vascular compromise caused by the neoplasm (4). Follow up is essential to assure the lesion heals properly after the initial biopsy. Although fine-needle aspiration has been suggested as a diagnostic tool (14), a biopsy remains essential to obtain a representative sample of the affected area and rule out additional pathologies (2 , 4 , 13). A thorough patient history-particularly regarding potential etiologic factors and lesion duration-combined with clinical and histopathological evaluation is critical for accurate diagnosis. Follow-up is equally important to confirm lesion resolution, which typically occurs within six weeks (3). This rapid healing contrasts with the behavior of most salivary gland neoplasms, which do not exhibit rapid progression or spontaneous resolution (2 , 13).

## Conclusions

In cases of swellings of the hard palate or in areas where salivary glands exist awareness of NS as a potential diagnosis should be considered. Clinical history and appropriate biopsy extension are relevant to obtain an adequate diagnosis and discard neoplastic pathologies. Follow-up of the patient is important to corroborate the resolution of the lesion.

## Data Availability

The datasets used and/or analyzed during the current study are available from the corresponding author.
